# Humanitarian Aid Workers’ Mental Health and Duty of Care

**DOI:** 10.5964/ejop.v15i4.2221

**Published:** 2019-12-19

**Authors:** Liza Jachens

**Affiliations:** a Webster University, Geneva, Switzerland; Webster University Geneva, Geneva, Switzerland

## Abstract

Set in challenging and complex environments, there has been growing concern about the mental health consequences of aid work. Along with existing difficulties in reducing well-known occupational risks such as exposure to trauma, there is a lack of awareness of psychosocial risks in the humanitarian sector. This paper is a discussion, drawing on occupational health perspectives, on ways to reflect on mental health policies, research and interventions in this sector.

The context of humanitarian aid work is intrinsically demanding. The term “aid workers” refers to persons who, on a professional or voluntary basis, engage in activities that help people in complex environments (Sifaki-Pistolla, Chatzea, Vlachaki, Melidoniotis, & Pistolla, 2017). Facing ongoing problems associated with civil conflicts, poverty, famine, disease and natural disasters, humanitarian and development aid is designed to save lives, ease suffering and respond to ongoing structural issues, particularly systemic poverty. Aid workers may be directly exposed to threats of harm and persecution and indirectly exposed to the trauma of the communities they serve. It is not surprising then, that researchers have begun to consider how the wellbeing of aid workers might be protected and promoted. The existence of foreseeable risks places responsibility on those who can shape and influence occupational mental health policies, research, training and interventions.

A review of aid worker health highlights implications of exposure to stressful aspects of the job. A build-up of stressful experiences and/or trauma has been shown to be linked to depression, anxiety, burnout, heavy drinking, secondary traumatic stress, and posttraumatic stress disorder (Ager et al., 2012; Connorton, Perry, Hemenway, & Miller, 2012; Eriksson, Kemp, Gorsuch, Hoke, & Foy, 2001; Jachens, Houdmont, Thomas, 2016, 2019; Lopes Cardozo et al., 2013; Jones, Müller, & Maercker, 2006). Priority needs to be given to identify the work-related risk factors that are associated with such outcomes. If the sources of stress are known, this can inform the design and targeting of interventions to eliminate, reduce, or buffer exposure to potentially harmful work characteristics.

Stress research involving emergency services personnel, and by extension humanitarian aid workers, has mostly concentrated on the experience of traumatic stressors as the main predictors of employee wellbeing. The conceptual framework often used to understand complex humanitarian aid work is the paradigm of trauma and post-traumatic stress disorder (PTSD) (Thomas, 2008). However, while this medical model brings focussed attention to those in crisis, it is limiting the full understanding of workplace risk factors.

It can be argued that, by adopting this medical paradigm, humanitarian aid workers’ strengths, such as motivation and resilience, are sometimes overlooked. As an occupational group they demonstrate substantial resilience and may reap rewards from their work such as job satisfaction, personal meaning and improved wellbeing (McFarlane, 2003). Although resilience and protective factors remain a somewhat understudied area in this field, emerging research has suggested new ways to conceptualise resilience. For example, Nolty, Bosch, An, Clements, and Buckwalter (2018) have recently developed the Headington Institute Resilience Inventory, a psychometrically valid, multifactored self-report measure to identify dimensions of resilience for aid workers. The objective is to identify dimensions that could lead to tailored protective training exercises; a promising approach to inform the sustainability of humanitarian efforts in difficult operating environments. In McCormack & Bamforth’s (2019) study on the subjective experience of aid workers deployed to provide support during the Ebola epidemic, personal strengths such as moral and professional integrity, and a strong sense of their own altruistic purpose, were found to be protective against psychopathology. Aid workers are rewarded by fieldwork – they identify with the powerful and motivating mission of protecting and helping - factors that motivate and connect them to the work they do (Jachens, Houdmont, Thomas, 2018).

The medical model, focussed on pathology, places importance on the individual (to receive treatment), rather than on the organisation (to prevent and reduce work-related stressors) in the reduction of job strain. Organisations may be inclined to pay attention to individual employees and develop their coping skills, rather than address the organisational sources of work-related stress (Murta, Sanderson, & Oldenburg, 2007). However, a multi-faceted approach may be more beneficial.

Stressors can be divided into two categories: job content and job context stressors. Overall, emergency service work includes both operational/occupational stressors arising from “job content” (i.e. the aspects of work inherent in the occupation, e.g. exposure to trauma or working overtime), and organisational stressors arising from “job context” (i.e. aspects of the work environment arising from the structure of the organisation e.g. shift work, workload, unfair work practices, lack of support from superiors, and excess paper work). Indeed, there is growing research evidence to suggest that job context (or organisational) stressors are of greater concern in human service professionals and that they have an important and more powerful effect on individual psychological and physical health outcomes than “job content” stressors (Brough, 2004; Huddleston, Stephens, & Paton, 2007). Surprisingly, not much research to date in the humanitarian sector has focussed on organisational sources of stress.

Two theoretical models have dominated occupational health research on work-related stress and informed the content of many psychosocial risk assessment instruments: The Job Demand-Control (JDC) model (or the Job Strain – JS – model) (Karasek & Theorell, 1990), and the Effort-Reward Imbalance (ERI) model (Siegrist, 1996). These self-report measurement instruments facilitate researchers and practitioners in the assessment of workers’ exposure to potentially harmful combinations of working conditions such as high demands with low control, or high effort with low rewards. Given the complexity of work-related psychosocial factors, measurement would benefit from being based on a theoretical model, which is a useful tool for dealing with real-life complex phenomena in the workplace. Research reliably shows that exposure to these poorly managed psychosocial work characteristics is associated with negative psychological, physical, and behavioural health outcomes (Theorell et al., 2015).

The humanitarian sector may benefit from a psychosocial risk assessment approach that engages more with current epidemiological psychosocial work environment models (as listed above). The inherent risk with this is that these generic models may not capture all the stressors that may be unique to this occupational sector. Future research could develop and explore a hybrid risk assessment tool that draws from generic stress models while also including job- and context-specific stressors. In developing a model, qualitative research is particularly valuable, as it can provide a deeper understanding of stressors, strains, and coping behaviours not captured by predefined constructs. What may be helpful is a model that examines both demanding (workload, role ambiguity) and motivational (altruism, engagement, connectedness) facets of humanitarian aid work, and the outcomes that ensue.

After psychosocial risk assessment, a further priority concerns the need for organisations to implement a comprehensive approach to stress prevention/reduction by embracing both individual and organisational interventions. Organisational interventions could address some common humanitarian job context stressors: a lack of teamwork, poor management and difficult supervisors, as well as the perceived lack of organisational support (Young, Pakenham, & Norwood, 2018). Individual support strategies include services such as briefings prior to and after assignment and counselling.

A growing literature base suggests the vital importance of management and organisational support for the wellbeing of humanitarian personnel (Ehrenreich & Elliot, 2004; Jachens, Houdmont, & Thomas, 2018; McCormack & Ell, 2017). Supportive organisational policies and practices integrate factors such as training, employee engagement, incentives for health promotion and protection, and evaluation and surveillance (Sorensen et al., 2013). Associated with improved support is the organisation’s psychosocial safety climate (PSC) – when psychological health and safety in the workplace is highly valued and prioritized. This PSC is determined by the prevailing beliefs, attitudes and behavioural norms within an organisation (Hall, Dollard, & Coward, 2010). In a high psychosocial safety environment, managers show commitment for the prevention of work stress and promote employee psychological health. The content area of PSC comprises: (1) senior management support and commitment for stress prevention (2) management priority to psychological health and safety (3) organisational communication – listening to employee contributions and (4) organisational participation and involvement through, for example, employee representatives (Hall, Dollard, & Coward, 2010).

Organisational values and policies should serve to promote and facilitate protection, compassion and trust. In a recent humanitarian conference organised by the CHS (Core Humanitarian Standard) Alliance the topic was “Building Trust in People, in Organisations and in the Sector” (October 2019, Antwerp, Belgium). This mirrors similar topics on social media platforms: Humanitarian aid organisations would like to develop more empathy, trust, compassion, and respectful ways of working in safer environments. A worthwhile focus is to develop a healthier PSC climate across the humanitarian sector. Organisations wishing to identify all probable sources of stress affecting their employees need to broaden their understanding of how different sources of stress influence health and wellbeing. Practitioners need to consider both harmful and resilience pathways in developing relevant and integrated interventions that can reduce stress in a comprehensive way.
